# Taiwanese family members’ bereavement experience following an expected death: a systematic review and narrative synthesis

**DOI:** 10.1186/s12904-024-01344-3

**Published:** 2024-01-11

**Authors:** Hui-Ju Liang, Qian Xiong, Bader Nael Remawi, Nancy Preston

**Affiliations:** 1 Division of Health Research, Faculty of Health and Medicine, Lancaster University, Health Innovation One, Sir John Fisher Drive, Lancaster, LA1 4AT UK; 2 Lancaster Medical School, Faculty of Health and Medicine, Lancaster University, Health Innovation One, Sir John Fisher Drive, Lancaster, LA1 4AT UK; 3 International Observatory on End of Life Care, Division of Health Research, Lancaster University, Health Innovation One, Sir John Fisher Drive, Lancaster, LA1 4AT UK

**Keywords:** Bereavement, Family, Expected death, Taiwan, Narrative synthesis, Mixed-studies review, Chinese

## Abstract

**Background:**

Bereavement experience is shaped by cultural and social contexts. No systematically constructed reviews were identified to explore the bereavement experience for people who are influenced by Chinese culture valuing filial piety and mutual dependence. This review aimed to systematically review the bereavement experience of Taiwanese family members living in Taiwan following an expected death.

**Methods:**

MEDLINE, PsycINFO, CINAHL, China Academic Journal Database, and Chinese Electronic Periodical Services were searched with no date restrictions from inception to 20 October 2022. The methodological rigour of studies was assessed using Hawker’s appraisal tool. A narrative synthesis approach using Popay’s work was employed to synthesise the findings of the studies. Studies investigating Taiwanese family members’ bereavement experiences were included. We excluded papers studying bereavement through the death of a child.

**Results:**

Searches retrieved 12,735 articles (after de-duplication), 17 of which met the inclusion criteria and were included for synthesis: English [9] and Chinese [8], published between 2006 and 2021. The studies varied in quality with scores ranging from 22 to 33 out of 36. The studies differed in the relationship between participants and the deceased, the bereaved time frames, and the definitions of bereavement. Most studies focussed on family members of cancer patients receiving specialist palliative care. Three bereavement theories and four tools were used. Risk factors of bereavement outcomes included family members feeling less prepared for death and deaths where palliative sedative therapy was used. Protective factors were higher caregiving burden and longer caregiving periods. Four themes regarding Taiwanese bereavement experience were generated: multiple impacts of death; problem-based coping strategies; importance of maintaining connections; influential religious beliefs and rituals.

**Conclusion:**

Continuing the relationship with the deceased is a key element of Taiwanese bereavement experience and it is influenced by religious and cultural beliefs. Suppressing or hiding emotions during bereavement to connect with the deceased and maintain harmonious relationships needs to be acknowledged as culturally acceptable and encouraged by some religions in Taiwan. The findings could be potentially relevant for other Chinese populations, predominantly Buddhist countries or other East Asian societies. The role of preparing for death in bereavement outcomes is little understood and requires further research.

**Supplementary Information:**

The online version contains supplementary material available at 10.1186/s12904-024-01344-3.

## Background

Bereavement care service is an essential part of delivering palliative care [1–3]. Bereavement experience is shaped by cultural and social contexts [4–6]. The bereaved express their grief through social acts and mourning practices appropriate to their cultural context [7]. Hence, the provision of good bereavement support needs to consider the cultural and social context of the bereaved [5, 8], and this would enhance the quality of palliative care services.

Chinese is the largest ethnic group in the world, consisting of about 20% of the global population and numbering around 1.4 billion people worldwide [9]. The Chinese culture has been deeply influenced by Confucianism and collectivism, which values the importance of family, filial piety, mutual dependence [10], and concern for others [11]. Maintaining harmony in a group is also a dominant feature of the Chinese culture [12]. The experience of bereavement in Chinese culture has been explored using different research designs, including quantitative and qualitative. There is a wide range of previous systematic reviews on bereavement experiences such as abnormal grief [13–15], grief measurements [16, 17], interventions for bereavement [18, 19] and bereavement outcomes [20]. However, no systematically constructed reviews were identified to explore such experiences for people who are influenced by Chinese culture. A systematic review to synthesise the current evidence can allow a more comprehensive understanding to inform clinical practice regarding providing culturally sensitive bereavement care. Therefore, a review to synthesise the bereavement experience of Chinese family members should be carried out.

Although many areas of East Asia, such as Taiwan, China, and Hong Kong, share a similar traditional Chinese culture, the palliative health care systems are different. For instance, Taiwan was ranked in third place in the Quality of Death and Dying in 2021; China and Hong Kong were ranked 53rd and 23rd, respectively [21]. Given the differences in healthcare systems, a review to synthesise the bereavement experience of Taiwanese family members living in Taiwan under the context of expected deaths was planned. The review excluded unexpected deaths such as accidents, disasters, and suicides as they are associated with a more difficult bereavement, including greater posttraumatic stress disorder symptoms, enduring depression [22, 23] and abnormal grief [13, 24].

Western-oriented bereavement theories, such as Worden’s task model [25, 26] and the Dual Process Model [4, 27], were commonly employed to inform clinical practice in Taiwan. Western cultures tend to promote autonomy and individualism [28], which is very different from Chinese culture, as described earlier. The application of Western-oriented bereavement theories in Taiwan needs adaptation to consider cultural differences [5, 8]. Therefore, this review also aimed to inform which Western-oriented bereavement theories are more culturally appropriate in Taiwan and whether any new theories have been developed and applied in Chinese communities. The aim is these can inform future research and bereavement support practices for Taiwan and other Chinese populations.

## Methods

### The review question

What is the bereavement experience of Taiwanese family members following an expected death?

### The review design

An integrative review approach was used as it can include different designs to gain a wider understanding of the phenomena. The Enhancing transparency in reporting the synthesis of qualitative research (ENTREQ) statement [29] was followed in reporting the review.

*The search strategy.* Five electronic databases were searched from inception to 20 October 2022: MEDLINE, PsycINFO, CINAHL, China Academic Journal Database (CNKI), and Chinese Electronic Periodical Services (CEPS). A specialist health librarian was consulted for the search strategy. The following key concepts, along with synonyms and tailored subject headings, were used: ‘bereavement’, ‘family’, and ‘Chinese’ (Supplementary Material 1). The reason for using ‘Chinese’ as a keyword is that the majority of literature tends to define the population in Taiwan as Chinese (‘Hua-ren’, 華人). Boolean operators (AND, OR) and search commands such as truncation and proximity searches were applied [30]. There were no date restrictions. The MEDLINE database search string (Supplementary Material 2) was adjusted to other databases. Additional search strategies included: using key papers to test the searching strategy, searching Open Grey (grey literature database), screening reference lists of included studies, conducting citation tracking of included papers through Google Scholar, and setting alerts in MEDLINE, PsycINFO, and CINAHL to track potential new articles.

*Study eligibility.* Table 1 describes the inclusion and exclusion criteria. In the review, Taiwanese family members mean Taiwanese living in Taiwan as this review investigates the bereavement experience which is influenced by cultural and social contexts. Papers about people bereaved through the death of a child were excluded because it is recognised as a more challenging experience than other types of bereavement [31] and may have a higher risk for suffering in abnormal grief [32]. The main researcher (HJL) screened titles and abstracts of all retrieved articles, while a second reviewer (BNR) screened 10% of them to enhance the rigour of the screening process [29]. Disagreements of four articles (0.3%) were resolved through further discussions about the inclusion criteria among authors.

**Table 1:** Inclusion and exclusion criteria

	Inclusion criteria	Exclusion criteria
Study population	• Taiwanese over 18 years old living in Taiwan • Participants are family members or significant others of the deceased • The relationship to the deceased: spouse, parents, adult children, sibling, significant other, partner	• The bereavement caused by the death of a child
Study topic	• Studies focused on the experience of having lost a relative or significant other from an expected death, trying to adapt to the relative’s or significant other’s death or the process of grieving and mourning	• Studies focused on developing bereavement measurements or interventions • Unexpected death such as COVID-19, violence, accidents, disaster, suicide, and murder
Type of evidence	• Primary research • Peer-reviewed journal articles	
Language	• English, traditional Chinese	

*Quality assessment and data extraction.* Hawker et al.’s data extraction form [33] was adapted according to the review’s purpose to collect relevant data (Supplementary Material 3). For qualitative research, relevant descriptions of key themes were drawn from the included papers. Quantitative data which answered the review question was transformed into a textual description [34] as this review aims to explore the bereavement experiences such as adaptation after death.

Hawker et al.’s critical appraisal tool with a score ranging from 9 to 36 was chosen as it is suitable for reviewing different designs [33]. Assessment of study quality was conducted by the main researcher (HJL) while extracting data from all identified papers.

*Data synthesis.* Popay et al.’s approach to narrative synthesis was undertaken in an iterative manner [34]. Tabulation was conducted to summarise details of participants and key findings and to identify patterns and differences across studies. Translating data was applied to systematically identify the main themes that represented research findings. The results of the tabulation were imported into ATLAS.ti to assist with the translating process. Initial free coding was conducted line-by-line inductively across the studies. The researcher (HJL) grouped the codes and developed potential themes (Supplementary Material 4). Initial themes were renamed, and data were moved around to fit the themes throughout this process. These were reviewed and developed in an iterative process through discussion with NP and QX. A conceptual model was developed to visually present the findings through discussion (Supplementary Material 5). Finally, the dominant bereavement theories were used to interpret the review findings.

## Results

**Fig. 1:**
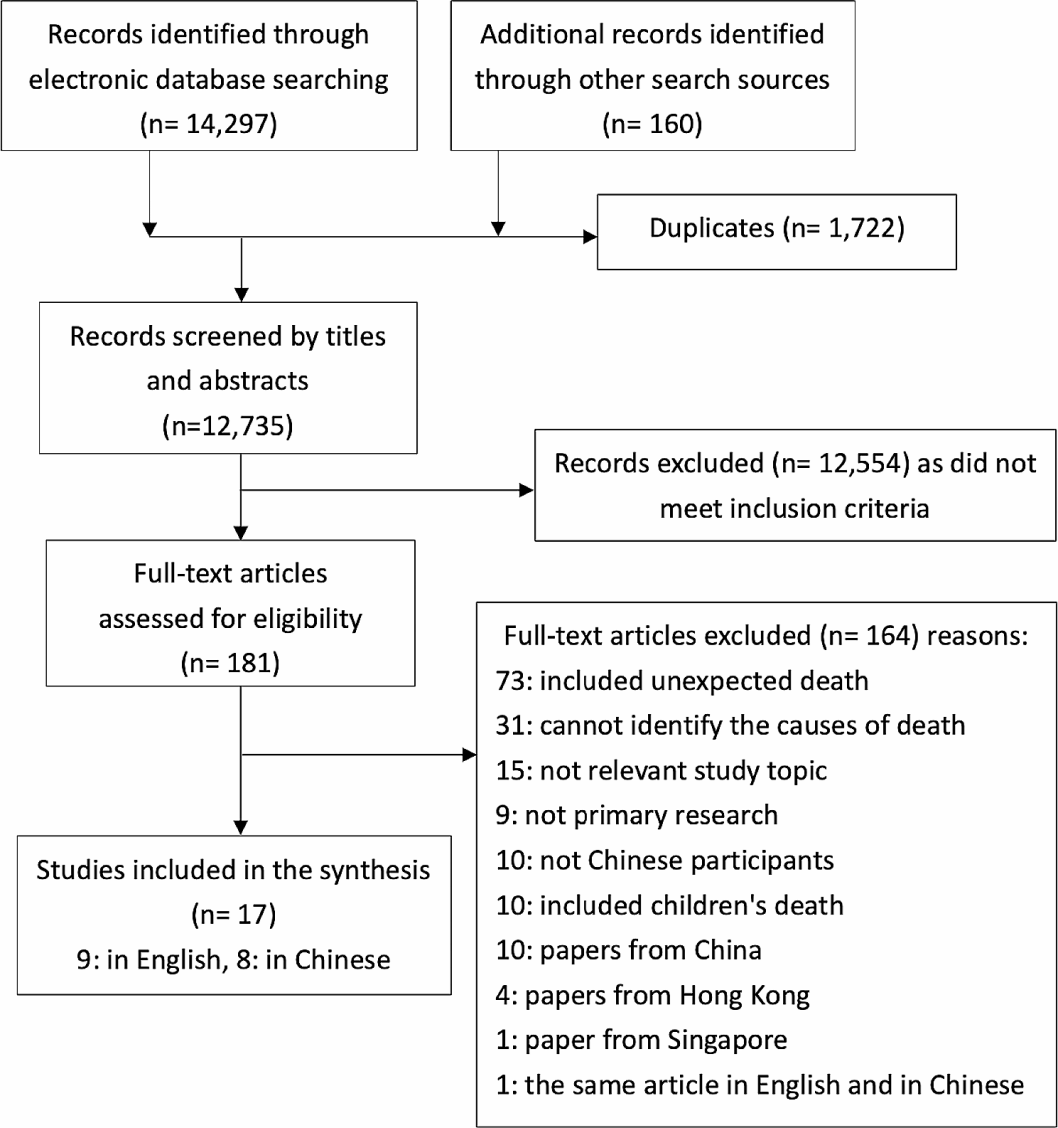
Search process flowchart (PRISMA flowchart)

Searches retrieved 12,735 articles (after de-duplication), 17 of which met the inclusion criteria of the review and were included for synthesis. The Preferred Reporting Items for Systematic reviews and Meta-Analysis (PRISMA) flowchart [35] shows details of the studies’ identification and selection process (Fig. 1).

### Study characteristics

The 17 papers were published between 2006 and 2021. There were ten qualitative studies [36–43, 45, 46], one mixed-methods study [47], and six quantitative surveys [48–53]. Six of the 17 studies employed longitudinal design [39, 43, 48, 49, 52, 53] (Table 2). The main aim of the qualitative studies was to explore family members’ bereavement experience, while the quantitative surveys mainly investigated the relationship between grief and specific variables such as family members’ demographics and palliative sedation therapy. Most studies (*n* = 12) recruited participants from a single hospital [39–43, 45, 46, 48–52], and two qualitative studies included only one family member as the participant [41, 42]. Studies were conducted across Taiwan. For an overview of the characteristics of the included studies see Supplementary Material 7.

The participants in the studies were 2,011 family members; nearly 65% were female. The mean age of family members was 46 years (range: 36–73). The majority of the participants held Buddhist beliefs, with some also practising Taoism/Daoism and Christianity. The included studies differed in the relationship between participants and the deceased and the bereaved time frames. The primary relationships were spouse and parent-adult children [36, 38, 46, 48–53]. The average bereaved time varied with the range between 36 hours and four years, but most studies were from six months to 18 months [37–39, 43, 45, 46, 50–52]. Most studies (*n* = 16) investigated terminally ill cancer patients [36–39, 41–43, 45–53] who received specialist inpatient palliative care (*n* = 10) [39, 42, 43, 45, 46, 48–51, 53]. No study investigated hospice home care patients. Table 2 describes the characteristics and the key relevant findings of the included studies.

The studies varied in quality with scores ranging from 22 to 33 out of 36; most studies (11 out of 17) scored between 26 and 32 [37–40, 43, 45, 47–51] (Supplementary Material 6). Among the nine domains of Hawker’s appraisal tool, the included studies were assessed as good or fair in the abstract and title, method and data, and results section. The common reasons for achieving poor or very poor quality in the included studies were related to ethics and bias [36, 37, 39, 49, 50], data analysis [36, 38, 41, 47], and implication and usefulness [36, 37, 39, 42] because of the limited information provided by authors.

**Table 2:** Characteristics and key findings of the included studies

Author, year of publication, Region	Participants/ Setting	Method	Key findings	Hawker score
Tsai, 2007, Middle Taiwan [36]	14 bereaved family members of cancer patients, the gender of the participants not mentioned	Grounded theory	1. The benefits of religion for family members’ bereavement: providing support, relieving shocks caused by the death of a loved one, religious communities became a supportive system, performing religious rituals which might be beneficial for the deceased, knowing the place afterlife the deceased has gone to, having continuing bonds with the deceased, believing in a reunion with the deceased in the future	22
Tsai, 2009a, Not mentioned [37]	11 bereaved adult children whose parents died from cancer (*n* = 7 females), recruited from family support groups or hospitals	Grounded theory	1. The deceased parent became virtual existence and had a new position with different functions in their family. The functions of the new position of the deceased included: communication, decision-making, and having space and affections such as feeling beloved by the deceased parent 2. The methods of continuing bonds between the bereaved children and the deceased parent included: fulfilling the deceased’s last wishes, carrying on the deceased’s legacy such as recognising the deceased as a role model	26
Tsai, 2009b, Not mentioned [38]	14 bereaved family members of cancer patients (*n* = 9 females), were recruited from family support groups or hospitals	Grounded theory	1. Changes in the family relationships of the bereaved after the death of a loved one included: becoming closer in family relationships, repairing conflicts among family members, becoming more isolated in family relationships, arguing with each other more often	26
Lin et al., 2011, Southern Taiwan [39]	10 bereaved caregivers whose spouse died from cancer and received specialist inpatient palliative care (*n* = 7 females)	Phenomenological Longitudinal interviews	Themes: the imaginative rumination, such as a sense of the deceased’s presence; transformative symbol, such as keeping or throwing away the deceased’s belongings; the ethical relationship, such as keeping thinking of the marital relationship and building or refusing new relationships	27
Hung, 2013, , Middle Taiwan [40]	6 bereaved adult family members of patients who died from chronic disease (*n* = 4 females)	Ethnography	Effects of performing funeral rituals included: having no time to go through the grief, accepting the truth of the death of a loved one, suppressing individual emotions, facilitating expression of collective emotions, receiving support from other relatives, experiencing reconnection with the deceased	29
Cheng, 2016, Not mentioned [41]	1 middle-aged widow whose husband died from lung cancer	Thematic analysis	Anniversary or holiday reactions of the bereaved included: feeling sad, feeling sorry for the children, going to the tomb, avoiding happy people, easily getting irritated with relatives and friends, spending holidays with friends having a similar experience	25
Jung and Hung, 2017, Not mentioned [42]	1 middle-aged, single female whose father died from cancer and received palliative care service	Narrative inquiry	Themes: the grief reactions such as poor appetite, crying alone, and suppressing emotions in publics, missing a lot about the deceased such as talking to the deceased and watching audio records of the deceased, learning to change such as learning to become independent and cherishing families and friends, accepting the death of the loved one such as the belief in a reunion with the deceased	23
Lee et al., 2017, Southern Taiwan [43]	10 family members whose spouse died from cancer and received specialist inpatient palliative care (*n* = 7 females)	Phenomenological Longitudinal interviews Secondary qualitative data analysis [44]	Theme 1: a blurred boundary of life (Yang) and death (Yin): reuniting the deceased through different means such as perceived physical encounters, dreaming of the deceased and performing religious rituals; receiving blessings from the deceased; love never dies and yuan (緣) never ends Theme 2: the transformation of relational bonds between the bereaved and the deceased such as believing in reincarnation; reinventing the ethical bonds among family members such as reassigning roles and responsibilities	31
Liang and Lai, 2020, Southern Taiwan [45]	6 bereaved adult children of cancer patients who received specialist inpatient palliative care (*n* = 5 females)	Focus group Content analysis	Themes: physical and mental suffering such as poor sleep, loss of weight, and missing the deceased sorely; bittersweet emotions such as sadness, self-blame, and no regret due to good death; unreal feelings and fighting back tears such as a sense of unreality and crying alone; scene-evoked memories such as seeing the deceased’s belongings; self-reflection such as reconsidering life goals	32
Lai et al., 2021, Southern Taiwan [46]	16 family caregivers of cancer patients who received specialist inpatient palliative care (*n* = 11 females)	Thematic analysis	Themes: grieving in silence; taboo topics such as avoiding talking about the deceased; emotion hiding such as maintaining a superficial “okay”; asynchronous grief; relational tension such as comparing the intensity of grief to each other family	33
Shih et al., 2010, Northern Taiwan [47]	20 older females whose husbands died from chronic disease, recruited from the community administration offices in five districts	Mixed method (survey and critical thematic analysis)	1. Participants with strong religious beliefs reported fewer coping problems 2. Coping problems the participants had, for example, loneliness, being withdrawn, low self-esteem, not wanting to become a burden to their children, low income, lacking help in housekeeping, moving home 3. Coping strategies the participants used, for example, learning self-care, making money, shopping by themselves, living a simple life, paying attention to their own health, receiving support from family members and friends, helping others, becoming optimistic, confident, and calm, praying, chanting, worshipping ancestors, searching for divination resources	29
Liu and Lai, 2006, Northern Taiwan [48]	120 adult family caregivers of terminally ill cancer patients who received specialist inpatient palliative care, 65% female	Longitudinal survey	1. The relationship between anticipatory grief and grief during bereavement remains unclear 2. Age and gender of family caregivers and their relationship to the deceased were not associated with grief during bereavement	28
Hsieh et al., 2007, Northern Taiwan [49]	46 family caregivers of advanced cancer patients who received specialist inpatient palliative care, 56.5% female	Longitudinal survey	1. There was no difference in grief reactions 1 month after death between family caregivers whose patients died at home versus those who died in the hospital 2. Predictor of grief reactions immediately after the death of the patient was the family caregiver’s educational level 3. Predictors of grief reactions 1 month after the death of the patient were the patient’s age and the perception that the patient had unfinished business	28
Chiu et al., 2010, Southern Taiwan [50]	668 bereaved family caregivers of terminally ill cancer patients who received specialist inpatient or palliative care consultation, 60.6% female	Cross-sectional survey	1. The prevalence of complicated grief was 24.6% (*n* = 164) 2. Risk factors of complicated grief: female gender, spouse relationship, parents-children relationship, no religious belief, unavailable family support, history of mood co-morbidity 3. Protective factors of complicated grief: longer duration of caring, caregivers with medical disease history, patients being cared for on the hospice ward	30
Chiu et al., 2011, Southern Taiwan [51]	432 bereaved family caregivers of terminal cancer patients who received specialist inpatient palliative care, 71.1% female	Cross-sectional survey	1. The prevalence of prolonged grief was 9.95% (*n* = 43) 2. Risk factors of prolonged grief: older age, female, spousal relationship, parent-child relationship, caregivers suffering medical disease 3. Protective factors of prolonged grief: education, higher income, a longer duration of caring for patients, religious belief, good family support, good social support	32
Tsai et al., 2016, Northern Taiwan [52]	493 family caregivers of terminally ill cancer patients in the general medical inpatient unit, 64.7% female	Longitudinal survey	1. The prevalence of prolonged grief among family caregivers of terminally ill cancer patients decreased through the first years of bereavement with 7.37% (28 out of 380), 1.80% (6 out of 334), 2.49% (7 out of 281), and 1.85% (4 out of 216) at 6, 13, 18, and 24 months after death, respectively 2. Risk factors of prolonged grief: caregivers who suffered from more severe depressive symptoms before the loss, perceived a more difficult dying process and death, and were less prepared for the death 3. Protective factors of prolonged grief: caregivers who reported higher subjective caregiving burden before death and perceived greater concurrent social support	33
Shen et al., 2018, Northern Taiwan [53]	143 family members of advanced cancer patients in a palliative care unit or terminal cases in six intensive care units, 55.2% female	Longitudinal survey	1. Family members of patients in the palliative care unit had lower grief levels than those in the intensive care units 3 days and 1 month after the death 2. For the palliative care unit, family members of patients who received palliative sedation therapy had higher levels of grief than those of patients who did not receive such therapy 3. Risk predictors of higher grief levels: good or very good intimacy relationship with patients, female family members, younger patients	33

### Exploration of definitions and theories of bereavement

The definition of bereavement varied among the included studies. Only three studies investigated complicated or prolonged grief [50–52]. Complicated grief was defined as the experience of having separation distress and post-traumatic stress and being unable to cope with the death [50]. Prolonged grief was described as experiencing intense grief reactions which lasted for more than six months [51, 52]. Moreover, Taiwanese researchers Lee et al. developed the term Bei-Dao (悲悼) [43]. Bei (悲) means individual grief; Dao (悼) indicates collective mourning and emphasises the continuing relationship with the deceased [43], which recognises the importance of relationships for the Taiwanese. Other studies also emphasised that reconnection with the deceased was vital for the bereaved [37, 40]. Two studies described bereavement as a family event and would impact the family dynamics [38, 46]; two studies illustrated that bereavement was the experience of making sense of the loss [39, 42]. The other four studies used diverse bereavement definitions: grief reactions with physical, psychosocial, and spiritual dimensions [45]; bereavement was related to the coping mechanisms of the bereaved and their support systems [47]; the bereaved might re-experience grief in a specific time such as the deceased birthday [41]; bereavement might impact the emotional and physical health status of the bereaved [49]. Three studies did not clearly define bereavement [36, 48, 53].

Three bereavement theories were mentioned: continuing bonds theory, Worden’s task model and meaning reconstruction theory [39, 42, 43, 46]. The continuing bonds theory represents a continuing relationship with the deceased and is a possible adaptive behaviour; Lee et al. considered such a relationship as vital for the Taiwanese [43]. Worden’s task model illustrates four grief tasks, such as acceptance of the loss, that should be achieved while going through bereavement. However, Lai et al. argued that the task of experiencing the pain of grief might not be suitable for the bereaved Taiwanese who tended to suppress and hide their emotions [46]. Two studies emphasised that finding meaning in loss is key, which was the important point of the meaning reconstruction theory [39, 42], but they did not provide further comments.

### Measurement tools

Four tools were used in six surveys (Table 3) [48–53]: the Chinese variation of the Inventory of Complicated Grief [50, 51] and the Prolonged Grief-13 [52] were used to detect abnormal grief; the Chinese version of the Texas Revised Inventory of Grief [49, 53] and the Chinese Perinatal Grief Scale [48] were used to assess the level of grief. The quality of the four tools was mentioned in all six surveys. Two studies explained that the Chinese variation of the Inventory of Complicated Grief was suitable for their research sample, supported by the literature [50, 51]. The Chinese version of the Texas Revised Inventory of Grief for family members of inpatient palliative care patients also demonstrated good psychometric characteristics [49]. However, this scale was then employed for terminal patients in intensive care units in the research of Shen et al. [53]. Finally, the Perinatal Grief Scale, initially developed for perinatal loss, was applied to family members of adult cancer patients in the work of Lit and Lai after the rigorous translation and validating procedures, including modifying to suitable wording and measuring internal consistency reliability for adult populations [48].

**Table 3:** Measurement tools of bereavement

Tool	Purpose	Scale items	Content subscales	Response format	Used in the included studies	
					Authors	Timing after death
Chinese variation of the Inventory of Complicated Grief	Detect complicated grief	19	No subscales Content: frequency of emotional, cognitive, and behavioural symptoms, E.g., anger over the death, avoidance of reminders of the deceased [54]	5-point Likert	Chiu et al., 2010 [50]	6–14 months Average: 8.9 months
					Chiu et al., 2011 [51]	6-14.2 months Average: 9.1 months
Chinese version of the Texas Revised Inventory of Grief	Assess reactions and levels of grief	26	3 subscales: 1) Past behaviours 2) Present feelings 3) An assortment of facts related to death	5-point Likert, True or false	Hsieh et al., 2007 [49]	1 month
		21	2 subscales: 1) Past behaviours 2) Present feelings	5-point Likert	Shen et al., 2018 [53]	3 days and 1 month
Prolonged Grief-13	Diagnose prolonged grief	Not applicable	No subscales The criteria include: 1) Experience of yearning 2) At least five of nine symptoms of functional impairment are caused by the death: E.g., feeling emotionally numb, stunned, that life is meaningless 3) Symptoms present more than at least six months after the death	Not applicable	Tsai et al., 2016 [52]	6, 13, 18, and 24 months
Chinese Perinatal Grief Scale	Assess grief during bereavement	33	3 subscales: 1) Active grief 2) Difficulty coping 3) Despair	5-point Likert	Liu and Lai, 2006 [48]	Approximately 2 months

### Predictors of bereavement outcomes

Six surveys investigated predictors of bereavement outcomes [48–53] (Table 4). All six studies included terminally ill cancer patients’ family members as participants; one of them also included family members of terminally ill patients in intensive care units [53]. Only one study included patients not in receipt of specialist palliative care [52].

**Table 4:** Protective and risk factors of bereavement outcomes

**Protective factors**	
Family members related	• Personal medical disease history [50] • Religious belief [51] • Education [51] • Reporting higher subjective caregiving burden just before patient death [52] • Longer duration of caring for patients [50, 51]
Patients related	• Being cared for on the hospice ward [50, 53]
Social related	• Higher income [51] • Good family support [51] • Perceived good social support [51, 52]
**Risk factors**	
Family members related	• Female [50, 51, 53] • Older age [51] • Educational level [49] • No religious belief [50] • Suffering their own medical disease [51] • Having a history of mood co-morbidity [50] • Suffering severe depressive symptoms before the death [52] • Good or very good intimacy relationship with patients [53] • The perception that the patient had unfinished business [49] • Perceived a more difficult dying process and death [52] • Less prepared for death [52]
Patients related	• Younger age [53] • Receiving palliative sedation therapy [53]
Other	• Spouse or parents-children relationship [50, 51] • Unavailable family support [50]

Some factors appeared modifiable through health care interventions before the death, including family members who felt that their loved one had unfinished business [49], felt less prepared for the death and perceived a difficult dying process and death [52]. Receiving specialist inpatient palliative care [50, 53] and Taiwanese family members having a faith were potentially beneficial for bereavement outcomes [47, 50, 51]. Furthermore, some predictors were somewhat counterintuitive. Higher caregiving burden [52] and longer caregiving periods [50, 51] could positively impact the bereavement experience. By contrast, receiving palliative sedation therapy was associated with higher levels of grief [53].

Although being female [50, 51, 53], of older age [51], and spouse or parents of children [50, 51] were the risk predictors for worse bereavement outcomes, one study by Liu and Lai showed that gender and the age of adult family caregivers and their relationship with the deceased were unrelated to the bereavement [48]. The two studies did not state whether lower or higher education was important [49, 51]. Overall, the quality of the six surveys was good; all scored over 28. A minority of participants in three surveys experienced complicated grief [50–52].

### Key themes

**Fig. 2:**
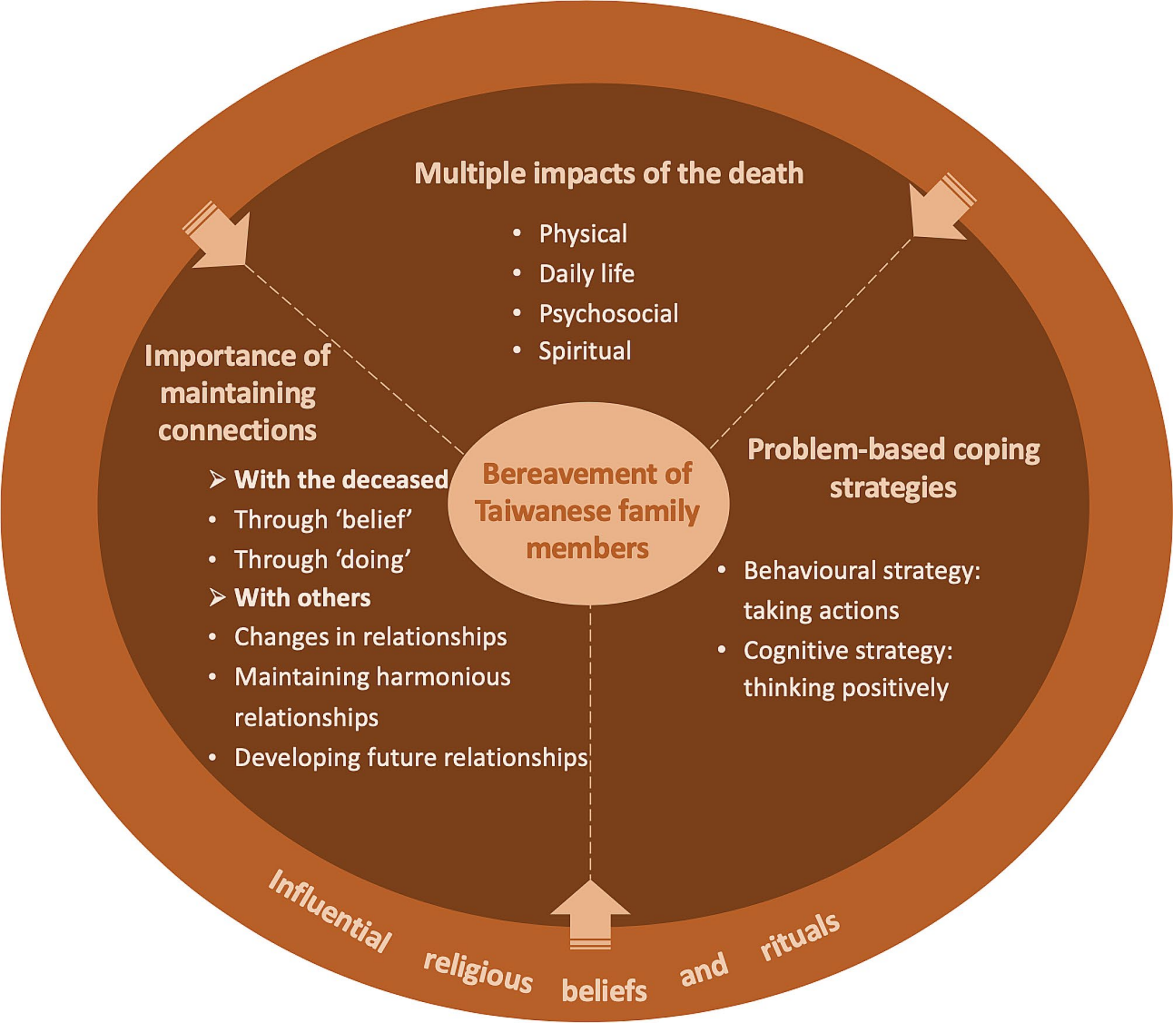
Themes of the bereavement experience of Taiwanese family members

Through the systematic comparison and exploration of the included studies, four themes about the bereavement experience of Taiwanese family members following an expected death were generated: multiple impacts of the death, problem-based coping strategies, importance of maintaining connections, and influential religious beliefs and rituals (Fig. 2).

#### Theme 1: multiple impacts of the death

The bereaved Taiwanese encounter multiple impacts caused by an expected death, including physical, daily life, psychosocial, and spiritual dimensions. The bereaved suffered from physical problems such as poor appetite [42] and poor sleep [45] which led to challenges in daily life, including lack of help in housekeeping and low income [47]. They also experienced considerable psychosocial impacts during bereavement, such as self-blame [40, 45], sadness [41, 45], missing the deceased [42, 45], worry about becoming a burden to others [47], and being irritated with other people [41]. However, the loss might offer them an opportunity to adjust their personal values and life goals by thinking of meaning in life [45].

#### Theme 2: Problem-based coping strategies

The bereaved Taiwanese tended to employ problem-based coping strategies to adjust to their life without the deceased, including the behavioural strategy - taking actions and the cognitive strategy - thinking positively. Taking actions indicated they learned new ways and changed their behaviours to cope, including learning self-care [47], engaging in personal religious communities, performing religious rituals [36, 40, 47], changing their caring focus to their children [39, 43], and reassigning roles and responsibilities amongst the family [43]. Additionally, thinking positively was a cognitive coping strategy the bereaved Taiwanese used which included trying to be confident, calm, and optimistic [47], cherishing families and friends [42], feeling happy because of having shared memories with the deceased, and having no regret due to good death of the loved one [45].

#### Theme 3: importance of maintaining connections

‘Importance of maintaining connections’ was the broader theme including two subthemes: continuing the relationship with the deceased and maintaining relationships with others.

*Continuing the relationship with the deceased.* Continuing the relationship with the deceased family member was particularly important. This was achieved through two means: ‘the belief’ and ‘the doing’. Regarding ‘the belief’, the bereaved Taiwanese believed a reunion with the deceased would happen in the future because of their religious belief [36, 42, 43]. Christians believed in meeting the deceased in Heaven [36, 42]. Buddhists believed in reuniting with the deceased following reincarnation. For instance, the deceased might be reborn as a new-born in their family [43]. The bereaved Taiwanese also believed in encountering the deceased in the future because of the concept of yuan (緣), a Chinese culture-specific belief which means the relationship is endless [43].

‘The doing’ was the second way of connecting with the deceased. Notably, the bereaved Taiwanese felt they did something beneficial for the deceased by conducting religious rituals such as chanting [36, 40], praying [36], and offering sacrifice [37, 41, 43] and not crying, because crying for the deceased would threaten the well-being of their soul [40, 46]. Maintaining or inheriting non-material resources related to the deceased was another vital connection. For instance, maintaining the deceased family member’s bloodline, life values, religious beliefs, and preferences such as singing songs they liked and also inheriting their roles and responsibilities in the family [37, 41, 42]. Additionally, there were several other ways of ‘doing’: physical encounters with the deceased such as seeing or hearing them, dreaming about the deceased [39, 43], keeping thinking of a time related to the deceased such as shared memories with them [39, 41, 45], talking to the deceased [37, 42]; and keeping and seeing physical materials which reminded them of the deceased such as their belongings, room, photos, and films [37, 39, 42, 43, 45].

*Maintaining relationships with others.* Maintaining relationships with others who were still living was vital too, including changes in relationships, maintaining harmonious relationships, and developing future relationships. Changes in relationships with others, because of the expected death, comprised positive and negative components. The bereaved Taiwanese might resolve conflicts with their family and become closer than before the death [38]. However, they might argue with each other more often and become more isolated with conflicted family relationships [38, 46]. This was because the death revealed or exacerbated existing family problems [38], or the bereaved could not understand and support their other family members [46].

Maintaining harmonious relationships was important for the bereaved Taiwanese but it required avoiding mentioning the deceased [46] and behaving well in public such as suppressing and hiding their emotions and crying alone [42, 45, 46]. Regarding future relationships, they might rebuild or refuse new relationships [39]; for instance, the bereaved spouse may decide to live in widowhood for their entire life [43].

#### Theme 4: influential religious beliefs and rituals

The final theme of the Taiwanese bereavement experience is influential religious beliefs and rituals. Religion was an important protective factor for bereavement outcomes [47, 50, 51]. The bereaved Taiwanese felt supported by their belief that they had a continuing relationship with the deceased [36, 40, 43]. Additionally, they felt comfortable knowing the place the deceased went after death such as Heaven (Abrahamic religions) [36, 42] and the Western Pure Land (Buddhism), where there is a world without suffering [40]. Performing funeral rituals according to religious beliefs also helped them accept the death, such as guiding the soul of the deceased family member to the paper spirit tablet which includes written the name of the deceased and symbolises the deceased’s soul [40]. However, they would suppress emotions and have no time to focus on their grief when conducting rituals and being with other people [40].

## Discussion

This review explores the bereavement experience of Taiwanese family members following an expected death. The results show that continuing the relationship with the deceased and suppressing or hiding emotions during bereavement deeply reflects the specific Taiwanese culture and the experience of family members. Importantly, the results show that the experience of family members before the death plays a vital role in their bereavement. Among the included studies, family members of cancer patients were the most common, possibly because cancer has been the leading cause of death in Taiwan for over four decades [55]. Cancer patients in Taiwan are more likely to receive specialist palliative care than people with noncancer. In 2017, 60.95% of cancer patients and 14.21% of noncancer patients received such care during their last year [56]. It is almost certain that specialist palliative care was the research context for most of the included studies.

### Continuing the relationship

This review suggests that continuing the relationship with the deceased family member is a vital and specific phenomenon in Taiwan. The idea of detaching from the deceased dominated the understanding of bereavement during most of the twentieth century, and this influential idea shifted about thirty years ago [57]. The review shows that the bereaved Taiwanese strongly believe they will reunite with the deceased in the future due to their religious and cultural belief, such as reincarnation and the concept of yuan (緣) [43]. The concept of yuan (緣), the belief that relationships are endless no matter death, is an influential opinion in Chinese society and a key notion of Buddhism as well. A likely explanation is that Buddhism is a powerful religion in Taiwan [58] although the participants’ religions in the review varied including Buddhists, Taoists, and Christians. Importantly, the review shows that the bereaved Taiwanese also believe they can help the deceased go to a better after-world through religious rituals [36, 37, 40, 41, 43]. Similarly, a study in Hong Kong showed that conducting rituals, such as burning paper, was believed to help the deceased have a better afterlife [59]. Indeed, caring about the afterlife of the deceased is a traditional Chinese value, as the bereaved believe the well-being of the deceased could influence their own life [60]. Such elements of the Taiwanese bereavement experience are very different from some Western experiences. Two studies in the United States investigating afterlife beliefs, religion, and bereavement adjustment did not show similar findings to this review [61, 62], including belief in reincarnation and improving the well-being of the deceased through rituals. Hence, the findings of the review may be appropriate for other Chinese populations or predominantly Buddhist societies.

The review found that ancestor worship is a culturally meaningful way to connect to the deceased in Taiwan [37, 41, 43]. The purpose of ancestor worship is to express filial piety, respect, and gratitude to the deceased senior family members [60]. This ritual has been widely performed by having tablets on a shrine in homes or communal ancestral halls across Taiwan [63] and many areas of East Asia due to the philosophy of Confucianism [60, 64]. Therefore, this review’s finding could also be potentially relevant to other East Asian societies and continuing the relationship should be understood in social and cultural contexts [65, 66].

The review shows that continuing the relationship may positively impact the bereavement by having ‘hope’ because of a belief in a future reunion with the deceased [36, 42, 43], and feeling comfortable knowing the deceased no longer suffers in their afterlife [36, 40, 42]. However, a study in Hong Kong showed some participants have negative feelings due to fear that the deceased went to hell [59]. Two studies from Western countries showed that continuing the relationship was associated with poor bereavement adjustments, such as depression [67, 68] and a higher level of grief [67], however, both employed different items to measure this variable [67, 68]. Consequently, whether continuing the relationship is beneficial or disadvantageous in coping with bereavement is still debated [26, 69, 70], possibly because its definition is complex and highly related to social and cultural contexts [65].

### Culturally acceptable and religious behaviour

The review shows that the bereaved Taiwanese avoid expressing emotions and tend to use behavioural and cognitive strategies to adjust to death. They suppress or hide emotions to avoid becoming a burden to others [46], thus, choosing ‘Bao xi bu bao you’ (報喜不報憂), which means to only report good or pleasant news, not bad news. Unlike the value of Western societies highlighting autonomy and individualism, Taiwan and other East Asian countries value mutual dependence and social relationships due to Confucianism [10, 60]. Hence, controlling negative emotions is key to maintaining harmonious relationships [71]. Suppressing or hiding emotions might also be helpful to maintain dignity which is highly valued in Chinese culture [5, 72, 73]. If a person cannot suppress or hide emotions properly, it may cause embarrassment that is knowns as ’shi tai’ (失態) and ‘diu lian’ (丟臉) [74], which means making a ‘gaffe’ and ‘losing face’, respectively.

The term Jie a.i. (節哀) is often used to express condolences for the bereaved in Chinese society. This expression comes from Confucianism, the book of Li Ji (禮記), which is a collective work by Confucian philosophers over two thousand years ago. The purpose of Jie a.i. (節哀) is to encourage the bereaved to restrain their grief, accept the death and move on with life because the death cannot be changed. From the perspective of Confucianism, expressing emotions is not an adaptative way to cope with bereavement compared to cognitive and behavioural strategies. It explains why the bereaved Taiwanese tend to employ problem-based coping strategies to adjust to death, as found in the review.

Moreover, suppressing or hiding emotions may be related to the religious beliefs of the bereaved Taiwanese. The review shows the bereaved chose not to cry for the deceased. This may be related to the predominant religion of Buddhism in Taiwan because Buddhists believe crying for the deceased would negatively influence their process of rebirthing to a better world and threaten their well-being in the after-world [58]. Similarly, a study in Hong Kong showed that family members tried not to cry at the moment of death as they worried it would affect the reincarnation of the deceased [75]. Consequently, the findings of the review seem to be suitable for other Chinese populations, predominantly Buddhist countries or other East Asian societies.

From the view of Western-oriented bereavement theories, it is widely believed that expressing emotions is a highly adaptive means of coping with bereavement [26, 76, 77]. Nonetheless, the review shows that it is unclear whether suppressing or hiding emotions negatively impacts Taiwanese bereavement. Two studies from outside of East Asian countries found that emotional expression was not beneficial for bereavement adjustments such as improvement in depressive symptoms [78]. Therefore, it may not be appropriate to simply emphasise the importance of expressing emotions in working through bereavement.

‘Emotional expressive flexibility’ may be more suitable for understanding the expression of emotions during bereavement. The concept means the ability to flexibly enhance or suppress emotional expression according to situational demands [79, 80]. As discussed before, the purpose of suppressing or hiding emotions for the bereaved Taiwanese is to maintain harmonious relationships [10, 60, 71] and avoid becoming a burden to others [11]. It highlights they value their interpersonal relationships when expressing emotions during bereavement. Studies from the United States show that expressing emotions according to individual and contextual needs was associated with better bereavement adjustment, such as fewer depressive symptoms [81] and less suffering from grief symptoms including self-blame and difficult acceptance of the death [82]. The role of emotional expressive flexibility in bereavement adjustment should be addressed in future bereavement research, especially in the context of Chinese culture.

### Reflections on Western-oriented bereavement theories

The continuing bonds theory may be more appropriate for understanding Taiwanese bereavement because continuing the relationship is very important. This theory was developed in the United States while working with bereaved parents and inspired by ancestor worship in Japanese culture [66, 83]. Nonetheless, ancestor worship has a very specific cultural meaning for showing respect to the deceased senior family members. Hence, this ritual is not an appropriate way of connecting with the junior deceased generations such as a child, as discussed before.

The continuing bonds theory proposes that the ongoing relationship with the deceased is normal and widespread, might be beneficial for bereavement adjustment, and should be understood in social and cultural contexts [57, 66]. The critical point of this theory is similar to the term Bei Dao (悲悼), developed by Taiwanese researchers [43]. The review shows that continuing the relationship deeply reflects Taiwanese cultural values and religious beliefs, including belief in reincarnation and yuan(緣), helping the deceased have a better afterlife, and not crying for the deceased. It supports the importance of assessing the religious beliefs of patients and their family members at the end of life. However, more work is needed to explore the application of this theory, especially in the context of Taiwan and other East Asia countries.

Other Western-oriented bereavement theories emphasising the importance of detachment from the deceased might not be appropriate for the Taiwanese who want to maintain the bond. For instance, John Bowlby’s popular attachment theory theorises that the development of affectional relationships early in life influences responses to the loss of a loved one. He proposed that the bereaved should detach from the deceased to recover from the loss [84].

Although some Western-oriented bereavement theories looked at bereavement from the view of continuing relationships or developing bonds, they seem to not be culturally appropriate for the Taiwanese. Worden’s task model, a widely used theory in clinical practice, proposes that finding a way to remember the deceased should be achieved [26]. The Dual Process Model, reporting the bereaved person oscillating between loss and restoration orientation coping behaviours to come to terms with the loss of a loved one, also emphasises maintaining an emotional bond with the deceased [4, 27]. However, both fail to mention that such a continuing relationship is related to cultural and social contexts, which is highly important for the bereaved Taiwanese based on the findings of the review. Worden’s task model suggests that working with the pain caused by the loss, such as sadness and anger, is one of the mourning tasks. Nonetheless, the review shows that the bereaved Taiwanese tend to suppress or hide emotions during bereavement to connect with the deceased and maintain harmonious relationships with others.

### Experiences at the end of life and bereavement

This review highlights the experience of family members at the end of life, including ‘Two P’ elements: patient care-related experience and preparedness-related experience, which could influence bereavement.

*Patient care-related experience.* The patient care-related experience includes (a) family members’ perceived quality of care leading up to the death of patients and (b) the caregiving experience of family members.

Firstly, this review shows that the bereaved Taiwanese may suffer from a higher level of grief if they perceive the patient had unfinished business [49] and a more difficult dying process and death [45, 52]. A study in the United States reported that better quality of death of cancer patients predicted better bereavement of family caregivers [85]. Nonetheless, palliative sedation therapy, a treatment for managing severe symptoms during end-of-life care, may negatively impact the Taiwanese’s bereavement [53]. Most family members participating in the study strongly agreed or agreed that there might be other means of relieving symptoms. A likely explanation is that they may worry that the most appropriate means to relieve patients’ suffering had not been used. It may also be related to Buddhism, as Buddhists believe that maintaining awareness during the process of dying is the key to rebirthing to a better world [58, 60]. To summarise, good quality of care leading up to the death of patients, especially good symptom management which maintains awareness, through appropriate interventions, may positively impact bereavement.

This review shows caregiving experience of family members at the end of life could also impact bereavement. The bereaved Taiwanese who had a higher subjective caregiving burden [52] and a longer caregiving period, may have better bereavement adjustment [50, 51]. This interesting finding reflects the Taiwanese culture emphasising a tendency for concern for those close to oneself [11]. Similarly, a systematic synthesis showed that family caregivers tend to ignore their own needs and feelings and do their best to relieve the patients’ suffering; they would be more satisfied with their caregiving experience because of a sense of fulfilling duty and showing love through care [86]. However, a qualitative study in China showed that family members perceived adverse caregiving, such as feeling exhausted, negatively impacted bereavement [87].

*Preparedness-related experience.* Preparedness-related experience of family members at the end of life, which would influence their bereavement, includes the experience of preparing for the death of a loved one. This review shows the bereaved Taiwanese could suffer from complicated grief due to less preparation for the death [52]. Similarly, two review articles from Europe showed that low levels of preparation for death were associated with abnormal grief [88, 89]. A Delphi study of developing a consensus on bereavement care in palliative care services in Europe highlights the importance of helping family members prepare for death and understand when death is impending [6]. Preparing for the death of family members seems beneficial for bereavement. Thus, ‘preparing for death and bereavement’ may be more appropriate for describing such an experience and it would be an essential issue in palliative care and end-of-life care. However, this topic is not well understood such as which components of preparing for the impending death impact the bereavement experience.

### Strengths and limitations

This is the first review to explore the bereavement experience of Taiwanese family members following an expected death. A systematic and comprehensive searching approach was used to gain a deeper and broader understanding, including using articles in English and Chinese and including different study designs (quantitative, mixed methods, and qualitative research). However, there are several limitations. Two qualitative studies included only one family member as the participant [41, 42]. Most included studies recruited participants from a single hospital (*n* = 12), investigated family members of cancer patients (*n* = 16), and explored the context of specialist palliative care (*n* = 10). Consequently, those may undermine the transferability of this review [90]. Although the review involved a second reviewer, the data extraction, quality appraisal and synthesis were conducted by only one reviewer, which might undermine this study’s rigour. However, some measures were taken to improve quality through discussions between authors throughout the study. Despite the limitations discussed here, the synthesis answers the review question, which reflects the specific bereavement experience of the bereaved Taiwanese.

### Future research

An alternative approach to exploring the topic of bereavement is necessary. There is an urgent need to investigate the experience of preparing for death and bereavement for family members and how this impacts bereavement. Future research should also focus on barriers to implementation for preparing family members for death and bereavement from the perspectives of healthcare providers. Moreover, for the bereavement theory development, continuing the relationship with the deceased is a relatively new notion and should be explored in social and cultural contexts. More work is needed to examine whether continuing the relationship with the deceased is beneficial for bereavement adjustment and to explore the continuing bonds theory, particularly in the context of Chinese culture. For instance, investigating the belief in reincarnation, which is a vital feature of continuing the relationship with the deceased in Taiwan and predominantly Buddhist societies, in coping with bereavement. The role of emotional expressive flexibility in bereavement adjustment should also be addressed in future bereavement research.

## Conclusions

The review suggests that continuing the relationship with the deceased is a key element of the bereavement experience for the bereaved Taiwanese and it is influenced by religious beliefs and cultural values, including the belief in reincarnation and yuan (緣), helping the deceased have a better afterlife by performing rituals and connecting to the senior deceased family members through ancestor worship. The continuing bonds theory could be useful for understanding the Taiwanese bereavement experience and potentially for people who are influenced by Chinese culture. Moreover, suppressing or hiding emotions during bereavement to connect with the deceased and maintain harmonious relationships needs to be acknowledged as culturally acceptable and encouraged by some religions in Taiwan. The review findings could be potentially relevant for other Chinese populations, predominantly Buddhist societies or other East Asian countries. More importantly, preparing for death and bereavement for family members would be critical at the end of life but it is not well understood, leading to a major obstacle to good palliative care and end-of-life care. Studies exploring the role of preparing for death in bereavement outcomes are required, aiming to improve bereavement care services [6].

### Electronic supplementary material

Below is the link to the electronic supplementary material.

**Supplementary Material 1:** Search terms

**Supplementary Material 2:** Example of electronic database searching (MEDLINE)

**Supplementary Material 3:** Data extraction form

**Supplementary Material 4:** Codes and initial themes

**Supplementary Material 5:** Drafts of a conceptual model

**Supplementary Material 6:** Result of quality assessment for the included studies

**Supplementary Material 7:** Overview of characteristics of the included studies

## Data Availability

All data generated or analysed during the study are included in this article. Additional data, including the search strategy, data extraction, quality appraisal and data synthesis, can be found in the Supplementary File and are also available from the corresponding author (h.liang3@lancasrer.ac.uk).

## Abbreviations

CEPS: Chinese Electronic Periodical Services
CINAHL: Cumulative Index of Nursing and Allied Health Literature
CNKI: China Academic Journal Database
ENTREQ: Enhancing transparency in reporting the synthesis of qualitative research
MEDLINE: Medical Literature Analysis and Retrieval System Online
PRISMA: Preferred Reporting Items for Systematic reviews and Meta-Analysis
