# Supplementary material for: Taiwanese family members’ bereavement experience following an expected death: a systematic review and narrative synthesis
Source: BMC Palliat Care. 2024 Jan 11;23:14. doi: 10.1186/s12904-024-01344-3 (PMC10782629; doi:10.1186/s12904-024-01344-3)
Supplement: Supplementary file 1 — Supplementary Material 1: Search terms [file 12904_2024_1344_MOESM1_ESM.docx]

**Supplementary material 1:** Search terms

| Concepts | MEDLINE | | PsycINFO | | CINAHL | | CEPS | CNKI |
| --- | --- | --- | --- | --- | --- | --- | --- | --- |
|  | MeSH terms | Title/Abstract | DE terms | Title/Abstract | MH terms | Title/Abstract | Title  /Abstract  /Keyword | Title  /Abstract  /Keyword |
| Concept 1  Bereavement | 1.Bereavement  2.Disenfranchised Grief | 1. bereav* 2. grief* 3. griev* 4. grief work 5. grieving process* 6. grief process* 7. mourn* 8. adapt* N4 loss 9. loss* | 1. Bereavement 2. Grief | 1. bereav* 2. grief* 3. griev* 4. grief work 5. grieving process* 6. grief process* 7. mourn* 8. adapt* N4 loss 9. loss* | 1.Bereavement  2.Bereavement Support (Saba CCC)  3.Personal Loss  4.Grief  5.Complicated Grief | 1.bereav*  2.grief*  3.griev*  4.grief work  5.grieving process*  6.grief process*  7.mourn*  8.adapt* N4 loss  9.loss* | 哀傷  (grieved)  悲傷  (grieving)  哀悼  (mourn)  悲悼  (mournful)  哀慟  (mourning)  悲慟  (grief)  悲痛  (grieve)  傷痛  (grievous)  喪慟  (mourning)  喪親  (bereavement)  悼念  (mourn) | 哀伤  (grieved)  悲伤  (grieving)  哀悼  (mourn)  悲悼  (mournful)  哀恸  (mourning)  悲恸  (grief)  悲痛  (grieve)  伤痛  (grievous)  丧恸  (mourning)  丧亲  (bereavement)  悼念  (mourn) |
| Concept 2  Family | 1. Family 2. Family Characteristics 3. Family Nursing 4. Family Relations 5. Family Separation 6. Military Family 7. Physicians, Family 8. Family Therapy 9. Family Practice   10.Family Conflict  11.Nuclear Family  12.Family Leave  13. Family Health | 1.Family  2.Families  3.significant other*  4.relative*  5.caregiver*  6.care giver*  7.carer*  8.next of kin  9.spouse*  10.conjugal*  11.kinship  12.widow*  13.grand*  14.parent*  15.father  16.mother  17.child*  18.daughter*  19.son*  20.sibling*  21.brother*  22.sister*  23.partner  24.individual*  25.people  26.person*  27.participant* | 1.Family  2.Biological Family  3.Dual Careers  4.Dysfunctional Family  5.Extended Family  6.Family Background  7.Family History  8.Family Members  9.Family of Origin  10.Family Relations  11.Family Resemblance  12.Family Structure  13.Family Work Relationship  14.Interethnic Family  15.Interracial Family  16.Military Families  17.Nepotism  18.Nuclear Family  19.Schizophrenogenic Family  20.Stepfamily  21.Family Relations  22.Structural Family Therapy  23.Family Work Relationship  24.Family Intervention  25.Family and Parenting Measures  26.Biological Family  27.Family Systems Theory  28.Strategic Family Therapy  29.Family Therapy  30.Family of Origin  31.Family Life Education  32.Family Background  33.Family Reunification  34.Schizophrenogenic Family  35.Family Work Conflict  36.Family Separation  37.Family Preservation  38.Family History | 1.Family  2.Families  3.significant other*  4.relative*  5.caregiver*  6.care giver*  7.carer*  8.next of kin  9.spouse*  10.conjugal*  11.kinship  12.widow*  13.grand*  14.parent*  15.father  16.mother  17.child*  18.daughter*  19.son*  20.sibling*  21.brother*  22.sister*  23.partner  24.individual*  25.people  26.person*  27.participant* | 1.Family  2.Extended Family  3.Professional-Family Relations  4.Physicians, Family  5.Family Relations  6.Patient-Family Relations  7.Family Systems Theory  8.Family Attitudes  9.Family and Medical Leave  10.Family Nursing  11.Nuclear Family  12.Dysfunctional Family  13.Military Family  14.Family Services  15.Family Integrity 16.Promotion 17.Childbearing Family (Iowa NIC) | 1.Family  2.Families  3.significant other*  4.relative*  5.caregiver*  6.care giver*  7.carer*  8.next of kin  9.spouse*  10.conjugal*  11.kinship  12.widow*  13.grand*  14.parent*  15.father  16.mother  17.child*  18.daughter*  19.son*  20.sibling*  21.brother*  22.sister*  23.partner  24.individual*  25.people  26.person*  27.participant* | 家屬  (family members)  照顧者  (caregiver) | 家属  (family members)  家人  (family)  重要他人  (significant other)  照顾者  (caregiver)  亲友  (relative)  亲戚  (relatives)  亲属  (next of kin) |
| Concept 3  Chinese | 1. Asian Continental Ancestry Group | 1. Chinese 2. China 3. Mainland China 4. Hong Kong 5. Macau 6. Singapore 7. Taiwan* | 1.Chinese Cultural Groups | 1.Chinese  2.China  3.Mainland China  4.Hong Kong  5.Macau  6.Singapore  7.Taiwan* | 1.Chinese | 1.Chinese  2.China  3.Mainland China  4.Hong Kong  5.Macau  6.Singapore  7.Taiwan* |  |  |
