# Supplementary material for: Taiwanese family members’ bereavement experience following an expected death: a systematic review and narrative synthesis
Source: BMC Palliat Care. 2024 Jan 11;23:14. doi: 10.1186/s12904-024-01344-3 (PMC10782629; doi:10.1186/s12904-024-01344-3)
Supplement: Supplementary file 2 — Supplementary Material 2: Example of electronic database searching (MEDLINE) [file 12904_2024_1344_MOESM2_ESM.docx]

**Supplementary material 2:** Example of electronic database searching (MEDLINE)

| Searching approach | Controlled search | Free-text search  (Limited to title or abstract) |
| --- | --- | --- |
| Keywords | Medical subject headings* | Key terms and synonyms |
| Concept 1:  Bereavement | Bereavement OR Disenfranchised Grief | bereav* OR grief* OR griev* OR grief work OR grieving process* OR grief process* OR mourn* OR adapt* N4 loss OR loss* |
| AND | | |
| Concept 2:  Family | Family OR Family Characteristics OR Family Nursing OR Family Relations OR Family Separation OR Military Family OR Physicians, Family OR Family Therapy OR Family Practice OR Family Conflict OR Nuclear Family OR Family Leave OR Family Health | family OR families OR significant other* OR relative* OR caregiver* OR care giver* OR carer* OR next of kin OR spouse* OR conjugal* OR kinship OR widow* OR grand* OR parent* OR father OR mother OR child* OR daughter* OR son* OR sibling* OR brother* OR sister* OR partner OR individual* OR people OR person* OR participant* |
| AND | | |
| Concept 3:  Chinese | Asian Continental Ancestry Group | Chinese OR China OR Mainland China OR Hong Kong OR Macau OR Singapore OR Taiwan* |
