# Supplementary material for: Taiwanese family members’ bereavement experience following an expected death: a systematic review and narrative synthesis
Source: BMC Palliat Care. 2024 Jan 11;23:14. doi: 10.1186/s12904-024-01344-3 (PMC10782629; doi:10.1186/s12904-024-01344-3)

**Supplementary material 3:** Data extraction form*

| Author, year | Objectives | | Participants | Setting | Design and Methods | Theoretical underpinning,  & Measurement, & Definition of bereavement | Relevant Findings/ Themes | Hawker Score |
| --- | --- | --- | --- | --- | --- | --- | --- | --- |
|  | |  |  |  |  |  |  |  |
|  | |  |  |  |  |  |  |  |
|  | |  |  |  |  |  |  |  |

*The form was adapted from Hawker et al. (2002) according to the purpose of this review.


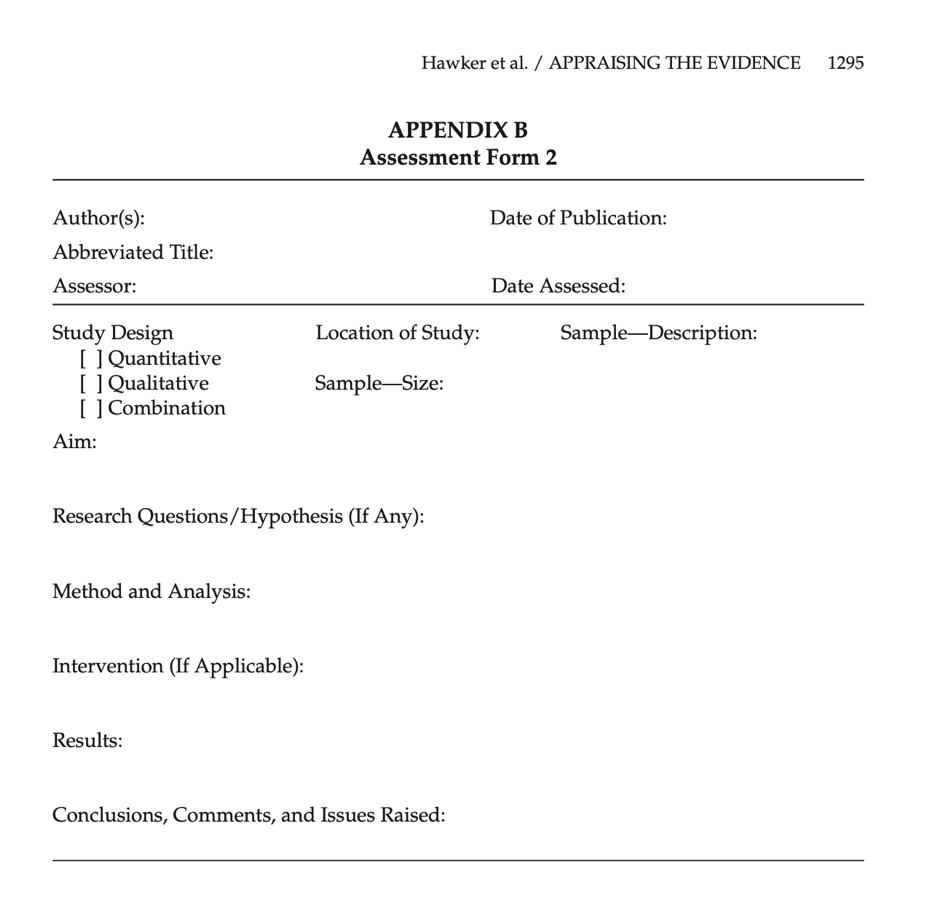

Supplement: Supplementary file 3 — Supplementary Material 3: Data extraction form [file 12904_2024_1344_MOESM3_ESM.docx]
