# Supplementary material for: Taiwanese family members’ bereavement experience following an expected death: a systematic review and narrative synthesis
Source: BMC Palliat Care. 2024 Jan 11;23:14. doi: 10.1186/s12904-024-01344-3 (PMC10782629; doi:10.1186/s12904-024-01344-3)

**Supplementary material 4:** Codes and initial themes


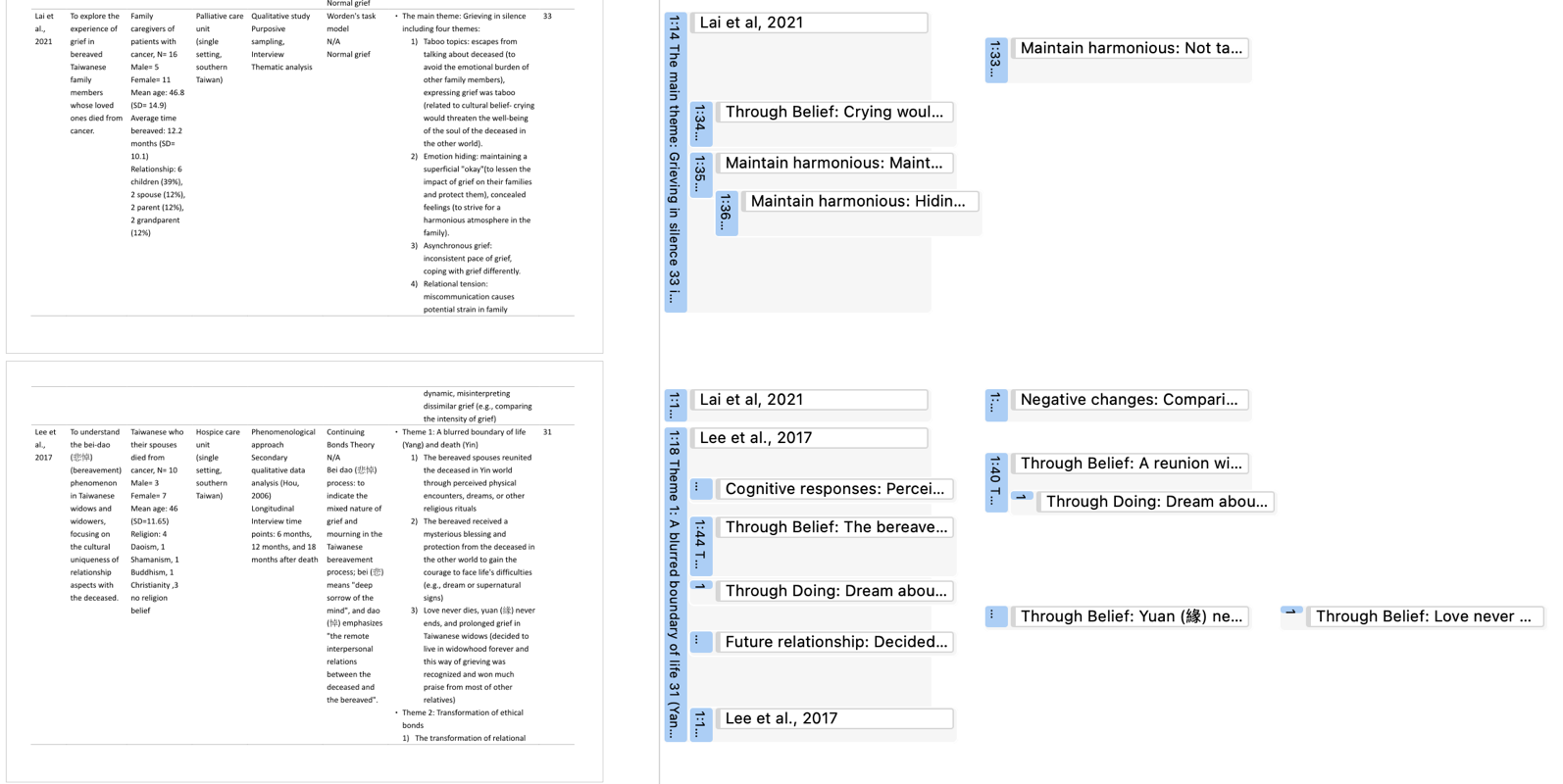


Screenshot from ATLAS.ti sued during the narrative synthesis process


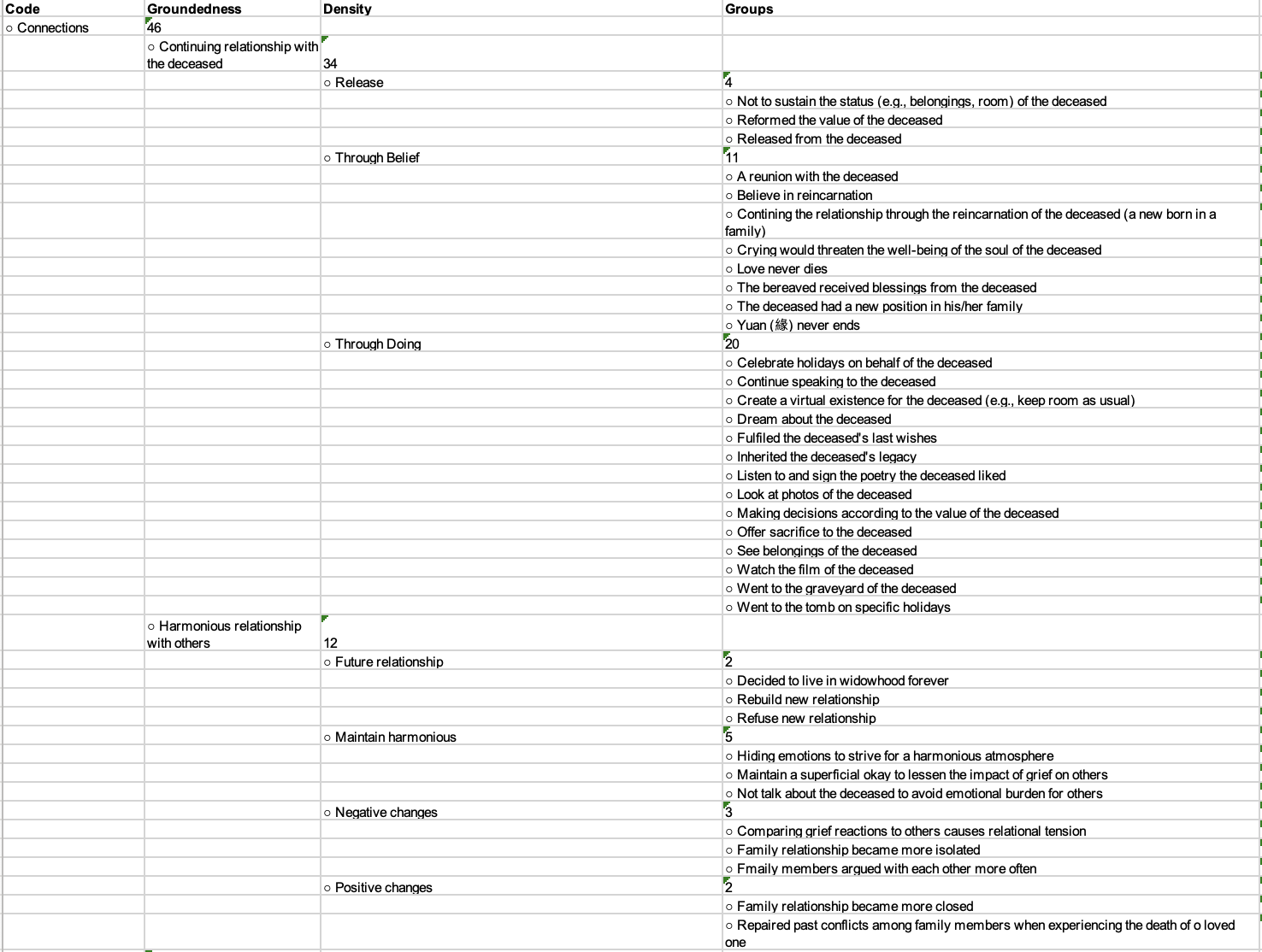


Screenshot from Code Manager of ATLAS.ti sued during the narrative synthesis process


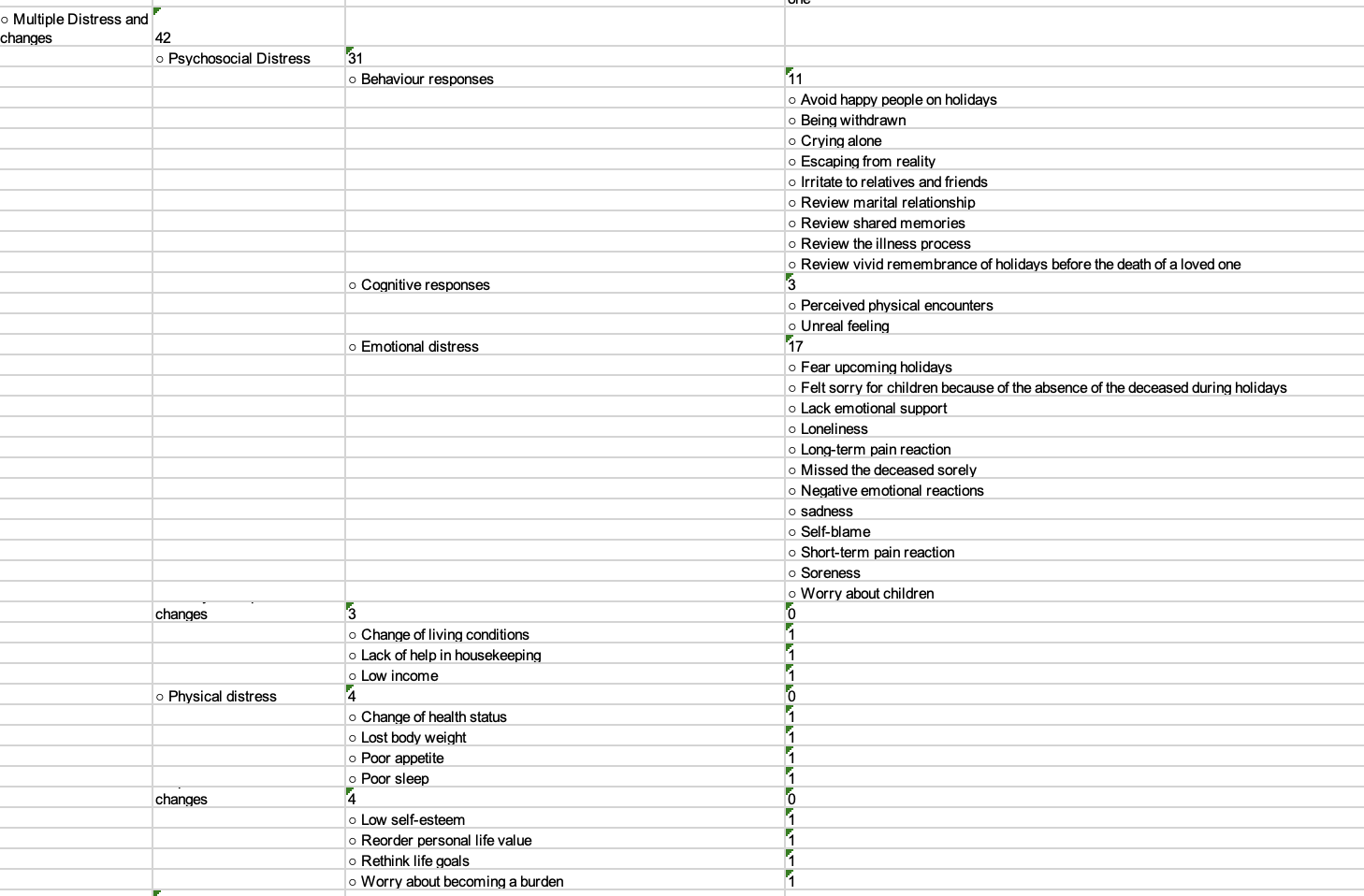


Screenshot from Code Manager of ATLAS.ti sued during the narrative synthesis process


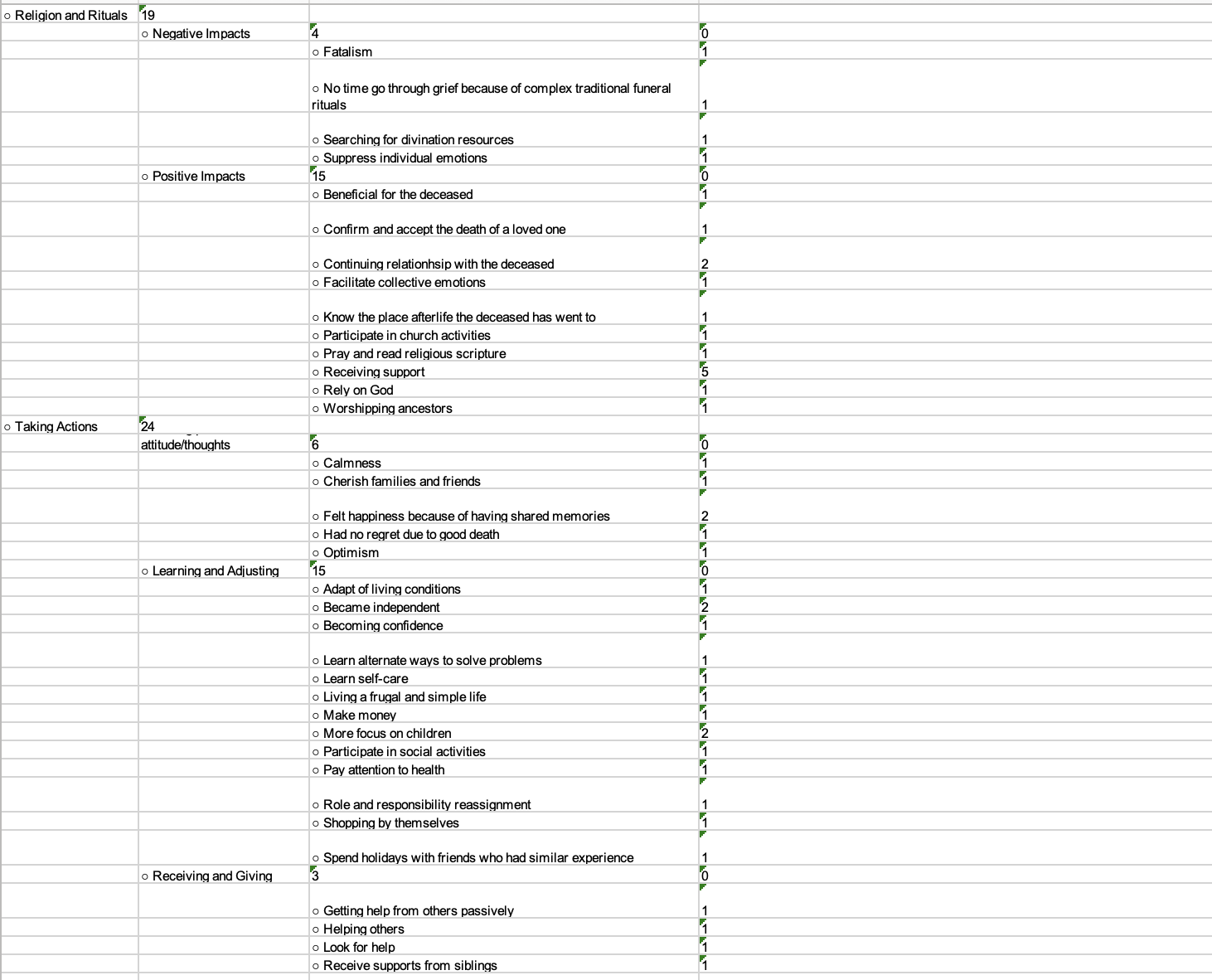


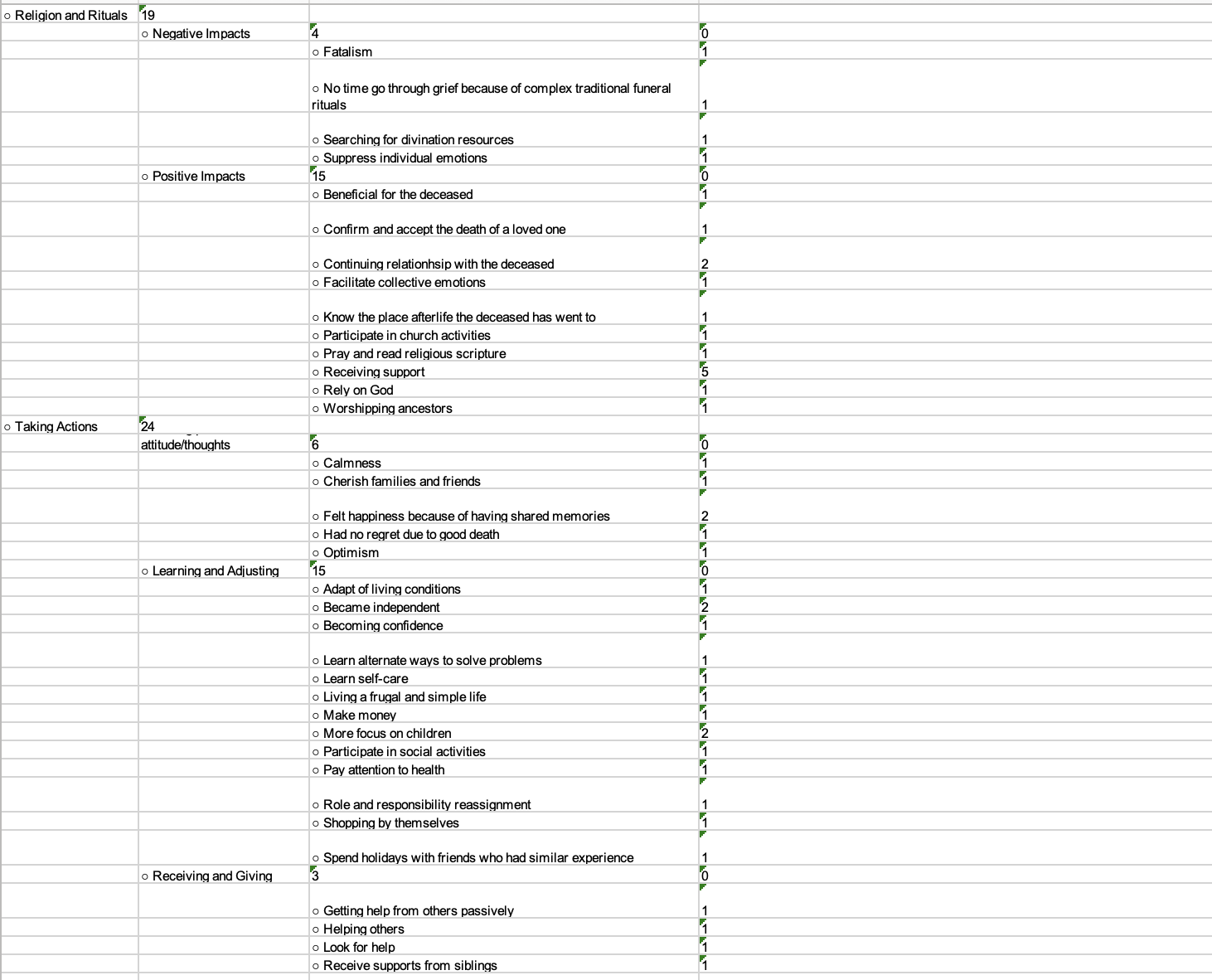

Supplement: Supplementary file 4 — Supplementary Material 4: Codes and initial themes [file 12904_2024_1344_MOESM4_ESM.docx]
