# Supplementary figures and images for: Taiwanese family members’ bereavement experience following an expected death: a systematic review and narrative synthesis
Source: BMC Palliat Care. 2024 Jan 11;23:14. doi: 10.1186/s12904-024-01344-3 (PMC10782629; doi:10.1186/s12904-024-01344-3)

**Supplementary material 5:** Drafts of a conceptual model

**
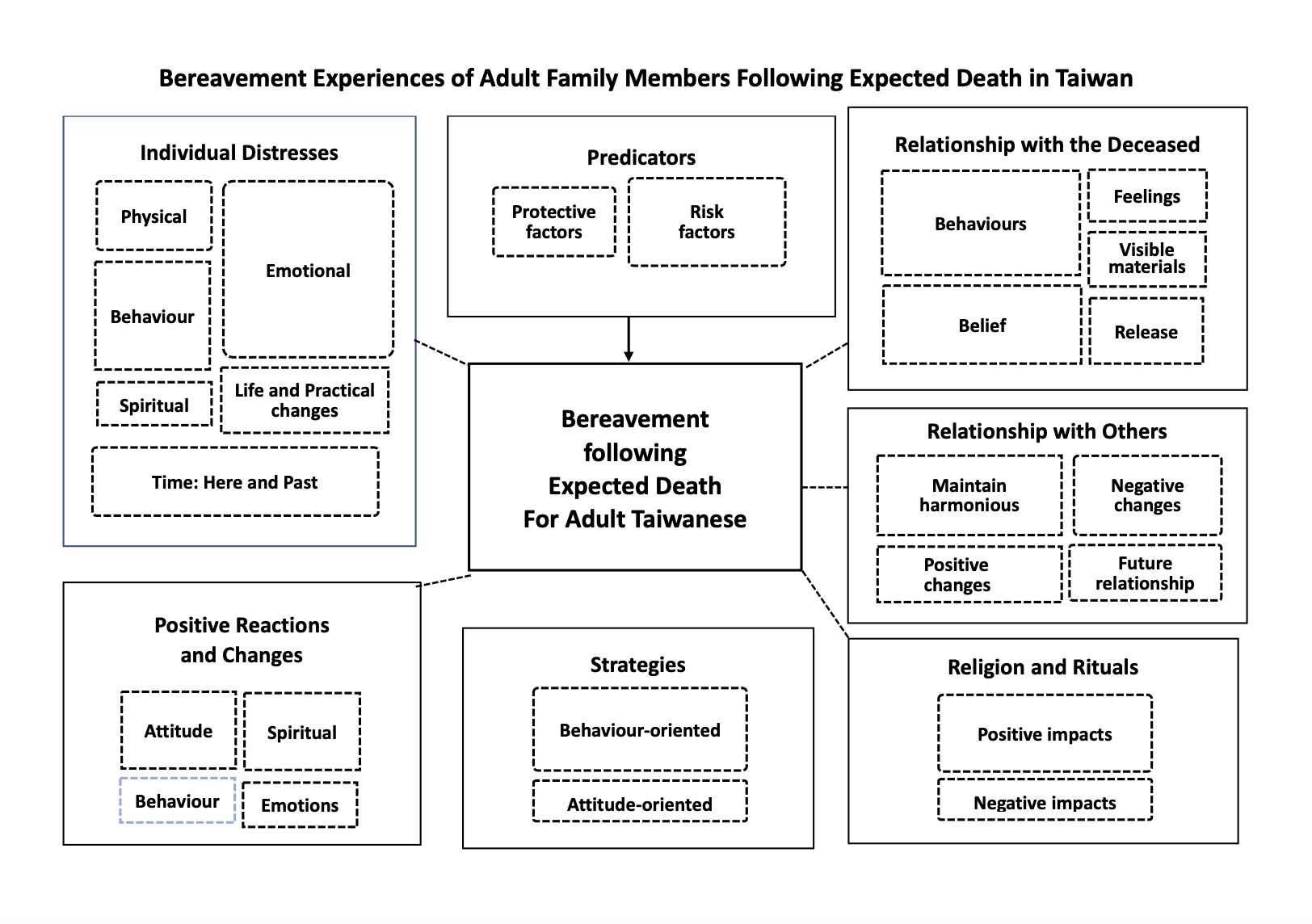
**

**
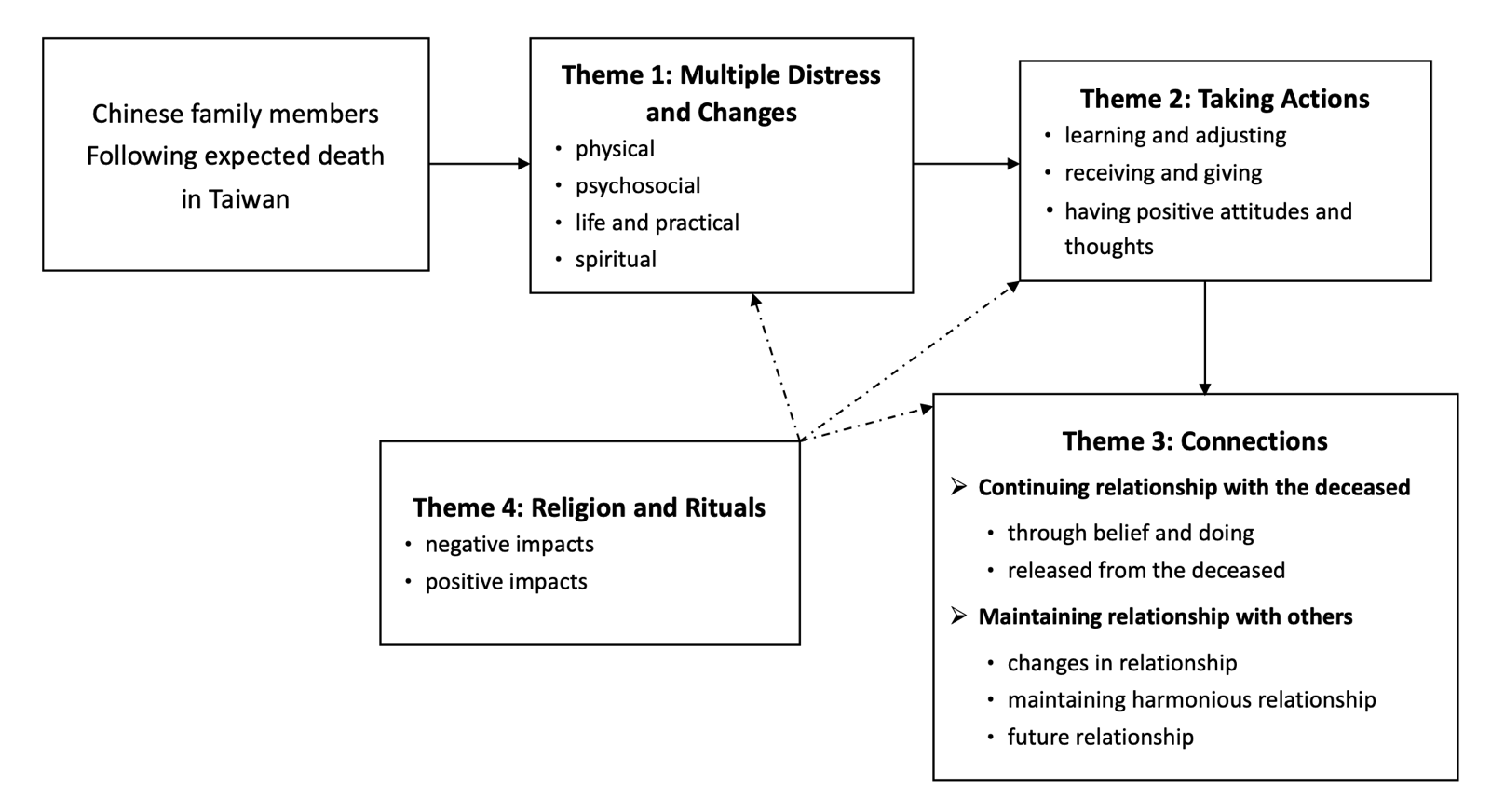
**

Supplement: Supplementary file 5 — Supplementary Material 5: Drafts of a conceptual model [file 12904_2024_1344_MOESM5_ESM.docx]

**Supplementary material 6:** Result of quality assessment for the included studies


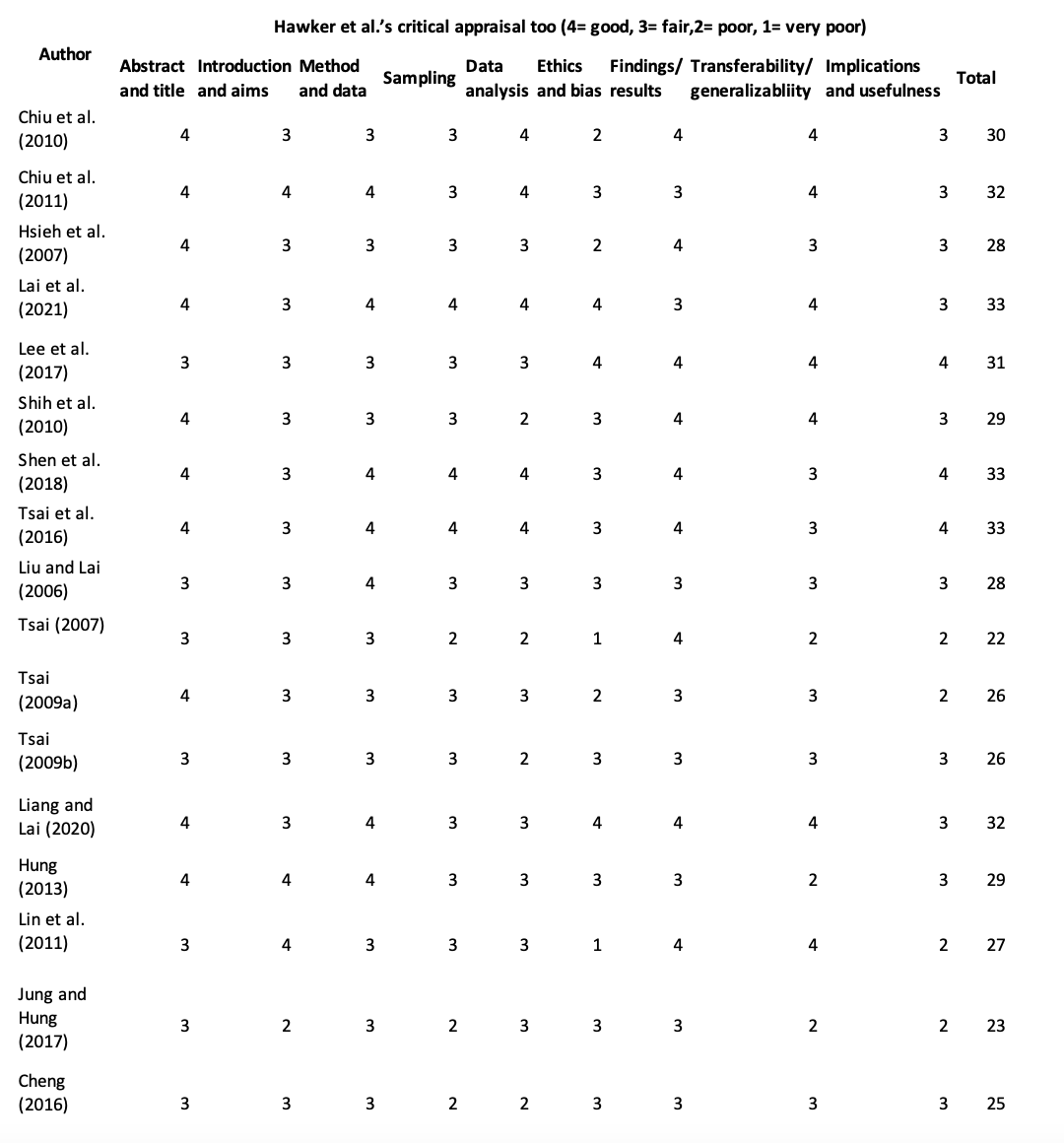

Supplement: Supplementary file 6 — Supplementary Material 6: Result of quality assessment for the included studies [file 12904_2024_1344_MOESM6_ESM.docx]
