# Supplementary material for: Taiwanese family members’ bereavement experience following an expected death: a systematic review and narrative synthesis
Source: BMC Palliat Care. 2024 Jan 11;23:14. doi: 10.1186/s12904-024-01344-3 (PMC10782629; doi:10.1186/s12904-024-01344-3)
Supplement: Supplementary file 7 — Supplementary Material 7: Overview of characteristics of the included studies [file 12904_2024_1344_MOESM7_ESM.docx]

**Supplementary material 7:** Overview of characteristics of the included studies

| **Reference** | **Participants** | | | | **Patents** | | **Design** | **Setting** | |
| --- | --- | --- | --- | --- | --- | --- | --- | --- | --- |
|  | Mean age  (years) | Religion  mentioned | Relation | Average time bereaved  (months) | Diagnosis | Palliative care |  | Region of Taiwan | Single hospital |
|  | 30-40  41-50  > 50  NR | Yes  No | Spouse  Parent-child  other | < 6  6-18  >18  NR | Cancer  Non-cancer | Yes  No  NR | Quantitative  Qualitative  Mixed  Longitudinal | Northern  Middle  Southern  Eastern  NR | Yes  No |
| Liu and Lai (2006) | X | X | X X X | X | X | X | X X | X | X |
| Hsieh et al. (2007) | X | X | X X X | X | X | X | X X | X | X |
| Chiu et al. (2010) | X | X | X X X | X | X | X | X | X | X |
| Chiu et al. (2011) | X | X | X X X | X | X | X | X | X | X |
| Tsai et al. (2016) | X | X | X X X | X X | X | X | X X | X | X |
| Shen et al. (2018) | X | X | X X X | X | X X | X | X X | X | X |
| Shih et al. (2010) | X | X | X | X | X X | X | X | X | X |
| Tsai (2007) | X | X | X X | X | X | X | X | X | X |
| Tsai (2009a) | X | X | X | X | X | X | X | X | X |
| Tsai (2009b) | X | X | X X | X | X | X | X | X | X |
| Lin et al. (2011) | X | X | X | X X | X | X | X X | X | X |
| Hung (2013) | X | X | X X | X | X | X | X | X | X |
| Cheng (2016) | X | X | X | X | X | X | X | X | X |
| Jung and Hung (2017) | X | X | X | X | X | X | X | X | X |
| Lee et al. (2017) | X | X | X | X X | X | X | X X | X | X |
| Liang and Lai (2020) | X | X | X | X | X | X | X | X | X |
| Lai et al. (2021) | X | X | X X X | X | X | X | X | X | X |

NR: not reported
